# Review—Recent Advances in Nanosensors Built with Pre-Pulled Glass Nanopipettes and Their Applications in Chemical and Biological Sensing

**DOI:** 10.1149/1945-7111/ab64be

**Published:** 2020-01-10

**Authors:** Megan Chang, Georgia Morgan, Fatima Bedier, Andy Chieng, Pedro Gomez, Sathya Raminani, Yixian Wang

**Affiliations:** Department of Chemistry and Biochemistry, California State University, Los Angeles, Los Angeles, California 90032, United States of America

## Abstract

Nanosensors built with pre-pulled glass nanopipettes, including bare or chemically modified nanopipettes and fully or partially filled solid nanoelectrodes, have found applications in chemical and biological sensing via resistive-pulse, current rectification, and electrochemical sensing. These nanosensors are easily fabricated and provide advantages through their needle-like geometry with nanometer-sized tips, making them highly sensitive and suitable for local measurements in extremely small samples. The variety in the geometry and layout have extended sensing capabilities. In this review, we will outline the fundamentals in fabrication, modification, and characterization of those pre-pulled glass nanopipette based nanosensors and highlight the most recent progress in their development and applications in real-time monitoring of biological processes, chemical ion sensing, and single entity analysis.

Glass nanopipettes fabricated by pulling borosilicate or quartz capillaries using a laser puller have become an essential tool for a wide range of analytical techniques.^1–3^ A nanopipette offers several important advantages including the ease of fabrication, small size, and needle-like geometry. This makes it a suitable probe for scanning probe microscopies, including scanning ion conductance microscopy (SICM) and scanning electrochemical microscopy (SECM).^4–9^ Nanopipettes have also been used for fundamental kinetics studies of charge transfer processes at the nanoscopic liquid/liquid interface^10–15^ as well as for resistive-pulse sensing, in which the ion current flowing through the nanopipette orifice is measured.^16,17^ A nanometer-sized particle can enter the orifice and partially block the current in a manner conceptually similar to the Coulter counter, as shown in Fig. 1a.^18^ The resulting spike in the current vs time curve (“resistive pulse”) can be used to detect the nanoparticle. Resistive pulse (RP) has been utilized for DNA detection and sequencing, particle separation, single-cell or organelle analysis, and other application.^17,19–23^

Modification of pre-pulled nanopipettes with different materials allows for a vast extension of their sensing capabilities. For example, selective sensing of ion transport can be achieved by coating the inside wall of a pre-pulled glass nanopipette with silanes, biorecognition molecules, or a conductive layer such as carbon.^16,24,25^ These modifications can manipulate the ion current rectification (ICR), which is described as asymmetry of the current-voltage response, i.e. the ion current at one potential polarity (+V) is much higher than at the opposite polarity (−V) for a potential of the same magnitude (Fig. 1b).^21,22,26–31^ Transport or binding of analytes to the inner wall can greatly affect the ICR, which in turn can be applied for sensing ions or molecules of interest.^16^

Depositing conductive materials, e.g., carbon or gold, into the pre-pulled nanopipettes brings electrochemical (EC) detection capability to the sensors.^7,24,32^ A typical example would be fully filling the nanopipette to form a solid disk nanoelectrode (Fig. 1c).^33–35^ Compared to traditional nanoelectrodes, which are fabricated by sealing metal into capillaries prior to pulling,^36–42^ deposited-nanopipette-based nanoelectrodes have a significantly thinner insulating wall, which dramatically decreases the overall size and thus are more suitable in electrochemical detection within small samples, such as individual cells, without destroying the cell membranes.^43–48^ These sensors can be applied to traditional electrochemical techniques such as cyclic voltammetry (CV), fast scan cyclic voltammetry (FSCV),^49–51^ and amperometry to detect analytes that are redox-active.^52^ Moreover, slight geometric modification of the solid nanoelectrodes allows for the design of new sensing devices. For example, a nanoelectrode with a small cavity at the orifice can be used for sampling ultra-small volume (attoliter-to-picoliter) of analytes and provide local amplification of the signal.^44^ The different sensing mechanisms—RP, ICR, and EC—can be combined by leaving an open channel within a nanopipette that is coated with a conductive layer inside.^28,53^ A special example, wireless nanoelectrodes (Fig. 1d), has also emerged as an effective alternative to traditional nanoelectrodes. By applying a high potential difference to an electrode, it is possible to polarize the unbiased electrode in order to utilize bipolar electrochemistry to generate redox reactions via ion flow instead of a metal wire. Bypassing the use of a metal wire avoids the background noise induced by gaps between the metal wire and the nanopipette inner wall, as well as the limited bandwidth and temporal resolution caused by the resistance and capacitance of the wire.^24^

In this review, we will outline the fundamentals in fabrication, modification, and characterization of pre-pulled glass nanopipette based nanosensors, and highlight the most recent progress in their development and applications in detection of real-time release of neurotransmitters and reactive oxygen and nitrogen species, ion sensing, and single entity analysis. Note that solid-state nanopores are similar to glass nanopipettes in some sensing capabilities but will not be covered in this review as the geometry and the fabrication process is completely different. Compared to solid nanopores, though nanopipettes are limited in the reproducibility, stability, and detection throughput, they are advantageous in the ease and low-cost of fabrication, and needle-like structure that allows localized detection in small volume. Discussions about solid state nanopores can be found in other reviews.^54–56^

## Fabrication, Modification, and Characterization of Nanopipettes-Based Sensors

The response of these nanopipette based sensors strongly depends on their size, geometry and chemical properties of the inside wall or filling materials. In the following section, we will briefly review the fabrication and characterization process of different sensors.

### Glass nanopipettes.—

Nanopipettes can be pulled directly from glass or quartz capillaries using a laser pipette puller, e.g., P-2000 produced by Sutter Instrument Co., with a careful selection of the material, thickness of the capillary wall, presence or absence of a filament, as well as the pulling parameters that will largely affect the final geometry of the pipettes. The details in parameter optimization can be found in our previous review.^16^ Prior to pulling, removal of metals and organic contaminants from substrates can be achieved by using a freshly prepared piranha solution, which should be handled very carefully at all times due to its explosive potential. After being treated with piranha solution, the glass capillaries should be rinsed thoroughly with distilled water and vacuum dried.^57,58^

Characterization of the pre-pulled nanopipettes is critical as the sensing capabilities, especially RP and ICR sensing, are highly dependent on the geometry. There are various approaches for characterizing the geometry of a nanopipette focusing on the two main parameters, the orifice radius (*a*) and the pipette angle at the tip (*θ*).^16^ Transmission electron microscopy (TEM) or scanning electron microscopy (SEM) provides the most direct measurements of both parameters with TEM offering a higher resolution.^59^ The short-comings are the high-cost of the instruments and possible damage of the pipette’s tip during sample preparation, preventing reuse of the pipettes after characterization. Alternatively, electrochemical methods—such as steady-state voltammetry of ion transfer at the interface between two immiscible electrolyte solutions (ITIES) at the nanopipette tip—can be used to evaluate the orifice radius from the diffusion limiting current (*i*_*d*_) expressed by *i*_*d*_ = 4*xz*_i_*FDca*, where *z*_*i*_, *D* and *c* are the charge, diffusion coefficient, and bulk concentration (in the external solution, e.g. 1,2-dichloroethane; DCE) of the transferred ion *i*, respectively; and *x* is a function of RG, which represents the ratio between the outer and inner diameters of a nanoprobe.^60^ This method provides relatively accurate measurement of *a* if a good steady-state voltammogram can be obtained. *θ* can then be determined by measuring the resistance of the system from a current-voltage (*i-V*) curve.^57,61^ The resistance is a combination of pipette resistance and solution resistance, and the former is related to *θ* and *a*.^16^

Modification of the inner wall of a pre-pulled glass nanopipette is critical for selective sensing of various analytes using the ICR effect and is typically done through covalent attachment, which provides stability especially with thiol-based linkages. A general approach is to back-fill the pre-pulled nanopipette with modification solution. For example, Pourmand et al. reported that the nanopipettes were backfilled with a 2% (v/v) (3-aminopropyl) triethoxysilane (APTES) solution that was prepared in anhydrous toluene, silanized for 1 h, and centrifuged at 4000 rpm for 15 min to remove bubbles. The nanopipettes were then washed from the inside with toluene to remove adsorbed APTES prior to the next modification steps of glutaraldehyde modification and covalent-attachment of a selective enzyme.^62^ After modification, the surface charge of the inner wall can be characterized using the *i-V* curve.

### Nanopipette modification with conductive materials.—

Modifying nanopipettes with conductive materials, e.g. carbon or gold, introduces the EC sensing capability, and controlling the deposition parameters can lead to variable types of nanosensors.^22,27,28,63,64^ In the case of depositing carbon through chemical vapor deposition (CVD), a carbon source, e.g., methane, together with a protector gas, e.g., argon, are passed through a pre-pulled quartz capillary at an appropriately high temperature. The flow rate, temperature and deposition time have to be carefully monitored to get desired results. With a shorter time (0.5 h) and lower temperature (875 °C), nanopipettes coated with a layer of carbon inside, or carbon nanopipettes (CNP), can be fabricated.^28^ The applications of those CNPs in sensing typically involve either recording ion current for RP and ICR sensing or CVs for EC sensing at the conductive carbon surface. With a longer time (>1 h) and higher temperature (>950 °C), it is possible to fully fill the capillary with carbon and thus make a disk-like solid carbon nanoelectrode (CNE).^32^ Platinization of CNEs is generally necessary for enhancing detection sensitivity, which can be done by etching a small cavity at the orifice of a CNE and depositing Pt black into the cavity through electrochemical seed-mediated growth.^35,47^

Similarly, a wireless nanoelectrode can be fabricated by using vapor deposition, such as via electron-beam evaporator or magnetron sputtering, to uniformly coat the inner wall of the nanopipette with a metal and using a hydrochloric solution to remove excess deposition on the outer walls. Alternatively, a gold nanotip can be fabricated by filling the nanopipette with AuCl_4_^–^ and reducing the solution with BH_4_^–^ until the nanopipette tip is filled.^24^ In the case of wireless nanoelectrodes, the metal part is not directly connected and biased. By using a pair of Ag/AgCl wires on either side of the pipette, ionic flow can be used to sense redox reactions at the interface of nanoscale metal deposits and the surrounding solution.^65^

Regarding characterization, TEM and SEM are again the straightforward ways of providing the details of the fabricated nanoelectrodes. Hu et al. utilized electron tomography to produce a 3-dimensional image of their pipette and found that this method yielded more accurate inner geometry of the electrodes.^46^ CV is also very useful in identifying the electrode radius, the existence of cavities, bipolar behavior, etc. A typical CV of a CNE with redox species should establish a sigmoidal shape with a plateau diffusion limiting current. A CNP or a CNE with small cavity would generate a voltammogram with a pair of essentially symmetrical peaks indicative of the thin layer cell type behavior.^44^

### Other facts about nanosensors.—

The reproducibility of nanoelectrodes on a mass scale while maintaining similar geometries utilizing these methods still proves to be difficult.^66^ A recent work from Wilde et al. uses in-situ TEM to real-time monitor the fabrication of carbon nanoelectrodes to achieve reproducible fabrication.^67^ Proper handling is required. These nanosensors can incur nanometer-scale damage by either electrostatic discharge (ESD), which occurs when electricity flows between two charged objects, or by electrochemical etching, which is the corrosion of metal by electrical current. To protect them from ESD, Nioradze et al. reported grounding the nanopipette during both experiments and storage using copper tape and maintaining the potential applied to the nanopipette by not disconnecting the electrode from the potentiostat between different measurements.^68,69^

## Application in In Situ Electrochemical Sensing of Neurotransmitters and Reactive Oxygen and Nitrogen Species in Cells and Tissues

Nanoelectrodes with small dimensions are suitable for real-time monitoring of release of electrochemically active molecules from cells and tissues such as neurotransmitters and reactive oxygen and nitrogen species (ROS/RNS), which have been extensively discussed in a few reviews.^32,34,37,70–73^ We will be highlighting a few novel works utilizing nanopipette based sensors from the last two years (Table I).

**Figure 2.**
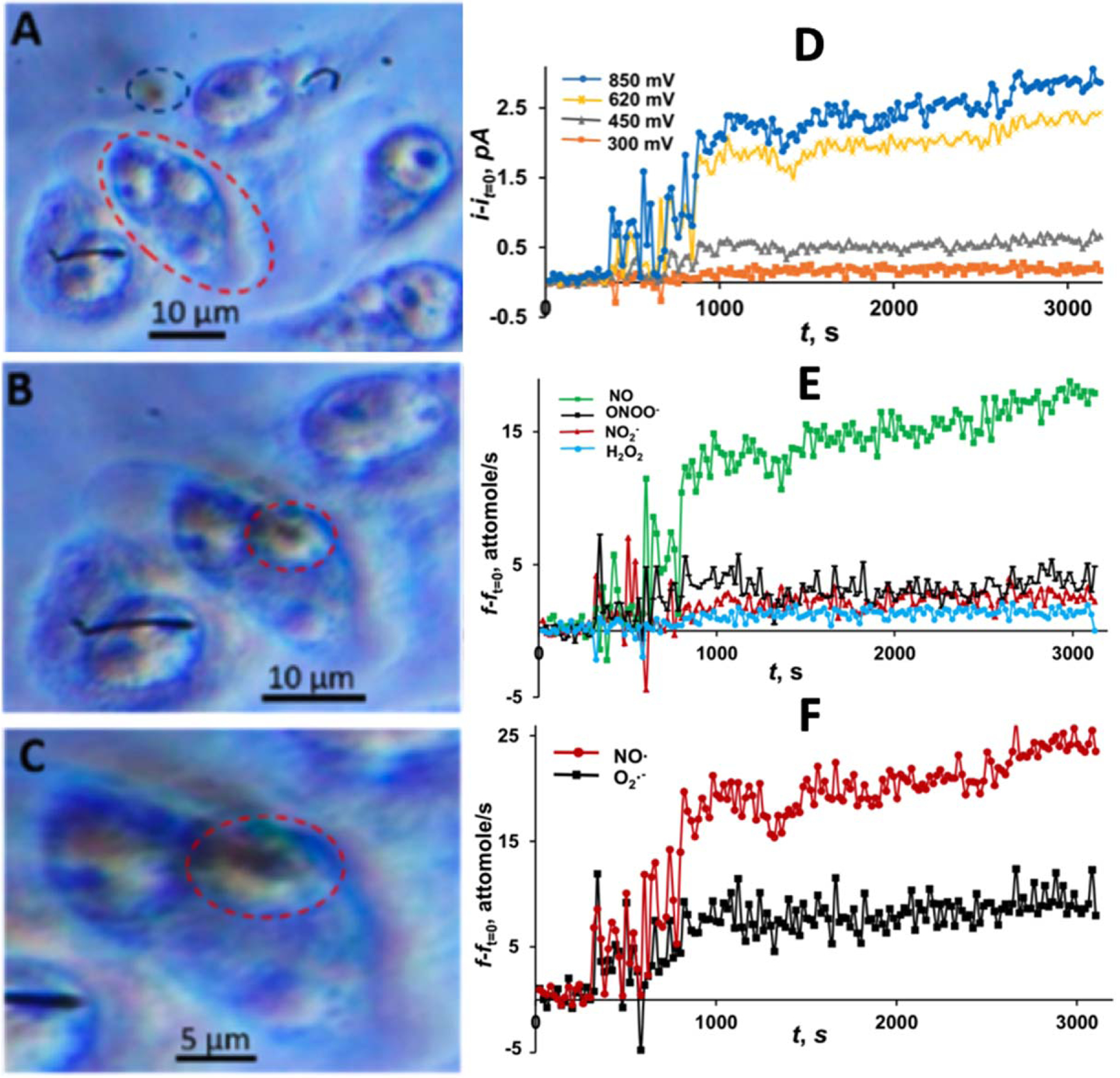
Optical images of phagolysosome penetration using a platinized nanotip shown (a–c); (a) the tip, shown in the blue circle, is close to the macrophage, shown in the red circle; (b) the nanotip is physically contacting the cell membrane above the targeted phagolysosome (red circle); (c) the nanotip is seen inside the phagolysosome. ROS/RNS measurements inside a phagolysosome of the activated RAW 264.7 macrophage (d–f); (d) time variations of chronoamperometric currents measured at different potentials at the tip inside a phagolysosome; (e) corresponding time variations of different ROS/RNS production rates calculated from currents in D and compared to its values at t =0; (f) time variations of the production rates of O_2_^·–^ and NO. Adapted with permission from Ref. 47 Copyright 2019 American Chemical Society.

Dr. Mirkin’s group used platinized CNEs smaller than 100 nm for quantifying intracellular production rates of four primary ROS/RNS (H_2_O_2,_ ONOO^–^, NO^·–^, NO_2_^–^) inside single phagolysosomes of living RAW 264.7 macrophages.^47^ ROS/RNS are molecules and ions that can cause oxidative damage to many living structures, including DNA, proteins, and tissues. In this work, approaching and penetrating individual macrophages and targeting single phagolysosomes were realized by using platinized CNEs as SECM tips with the guidance from an optical microscope (Figs. 2a–2c). Penetrated cells and phagolysosomes were able to maintain homeostasis as the membrane was able to reseal after penetration as the nanoelectrode had an inert shaft with a large aspect ratio (RG ≈ 1.5). Inside the phagolysosome, the time-dependent production of various ROS/RNS was detected by oxidizing them at different potentials. They were distinguished with quadruple potential-pulse chronoamperometry; it was observed that NO^·^ was the main species produced. Recording the amperometry at different oxidation potentials made it possible to extract the concentration of each species by serial-subtraction (Figs. 2d–2f). Overall, they speculated that the rapid oxidation of reactive oxygen and reactive nitrogen species at the nanoelectrode tip continuously stimulated ROS/RNS production to maintain high levels inside the phagolysosome for phagocytosis.

Selective detection of another ROS, superoxide (O_2_*^−^), in single living cells has also been achieved with a functionalized quartz nanopipette based on ICR sensing method.^62^ In this approach, the quartz nanopipette was treated with APTES, followed by modification with cytochrome c cross-linked with glutaraldehyde. The sensing principle is based on a redox process between the iron center of cytochrome c (i.e. Fe^3+^and Fe^2+^ ions) and superoxide radicals (O_2_ and O_2_*^−^). The current rectification is changed when Fe^3+^ accepts an electron to get reduced to Fe^2+^ and O2*^−^ oxidized to O_2_. The sensors showed high selectivity towards O_2_*^−^ only and did not respond to the presence of Mg^2+^, Cu^2+^, Zn^2+^, Ca^2+^, Fe^3+^, uric acid, glucose and ascorbic acid in the medium. The nanopipette surface was highly responsive towards O_2_*^−^ even after prolonged exposure to other species. Moreover, in situ analysis was realized by investigating the intracellular superoxide level in breast epithelial cells, which demonstrated the feasibility of this technique to studying single cancer cells for early diagnosis.

Venton et al. reported using cavity and open-tube CNPs (200 to 500 nm) to sample from nanometer-sized regions for neurotransmitter detection using FSCV.^45^ To test the CNP’s performance in tissue, dopamine was exogenously applied to mouse-brain slices. Open-tube CNPs were found to have slow temporal responses that change over time as the liquid rises in the CNP, while cavity CNPs have a fast temporal response, similar to traditional carbon-fiber microelectrodes.^51,82^ The cavity geometry enhances the electric field at the tip, allowing for a stronger electrostatic attraction for the positively charged dopamine during holding potential. Mirkin et al. further investigated those CNPs with smaller dimensions (5–200 nm).^46^ A detection limit of 100 pM was achieved for dopamine, as opposed to the previous 25 nM detection limit and the selectivity for dopamine was demonstrated by the ability to distinguish between dopamine and ascorbic acid.

To study *β*-glucosidase, a protein commonly found in lysosomes, Pan et al. used a nanopipette with the inner wall coated with platinum to detect hydrogen peroxide in the lysosome.^74^ *β*-glucosidase is involved in a series of reactions that occur inside of the liposome that lead to the release of hydrogen peroxide, thus the amount of hydrogen peroxide formed can be used to estimate the activity of glucosidase in the lysosome. Fluorescence was used to visualize the lysosomes and potential was applied at the electrode to guide the lysosome in before their membrane was permeabilized using Triton X-100 to isolate the glucosidase. Once the glucosidase was isolated from the lysosome, glucopyranoside and glucose oxidase were added to induce the release of hydrogen peroxide for detection. It was also discovered that lysosomes collected from the same cell exhibited very similar glucosidase activity while lysosomes from different cells exhibited different glucosidase activity.

Marquitan et al. developed a nanocavity-carbon electrode modified with polymer enzymatic films that can be used for amperometric sensing of glucose.^52^ These nanocavity-carbon electrodes (190 ± 25 nm diameter in pore size) were fabricated by etching carbon electrodes in 0.1 M KOH and then electrochemically modifying the pores with polymer-enzyme mixtures. Two different polymer-enzyme mixtures, poly(4-styrene sulfonate-co-glycidyl methacrylate-co-butyl acrylate-glucose oxidase and poly(1-vinylimidazole-co-allylamine) [Os(bpy)_2_Cl]Cl-pyrroloquinoline quinone-glucose dehydrogenase, were tested separately. During the CV analysis, the first type exhibited an oxidation peak of glucose and had a linear current response from 0 to 8 mM, while the second type exhibited an ideal sigmoidal-shaped voltammogram and had a linear current response from 0 to 15 mM. The detection limit was 0.13 mM and 0.016 mM, respectively. These polymer/enzyme carbon nanoelectrodes could eventually be used to measure glucose concentrations in vivo for living cells.

Note that most of the work has been done with nanoelectrodes in the case of detecting electrochemically active species as they can be easily oxidized or reduced and thus generate detectable signals. Detecting non-redox species electrochemically is difficult. Dr. Shen’s group recently proposed using a nanopipette to create a nano-ITIES between water and dichloroethane/octanoic acid inside the nanopipette to detect *γ*-aminobutyric acid (GABA). GABA is a neurotransmitter with a net neutral charge, meaning that it cannot be detected without some sort of modification. To enable electrochemical detection, GABA was protonated by lowering the pH of the solution with octanoic acid. After octanoic acid was added, the authors reported a linear increase in current as the concentration of GABA was increased.^75^

## Applications in Chemical Ion Sensing

**Figure 3.**
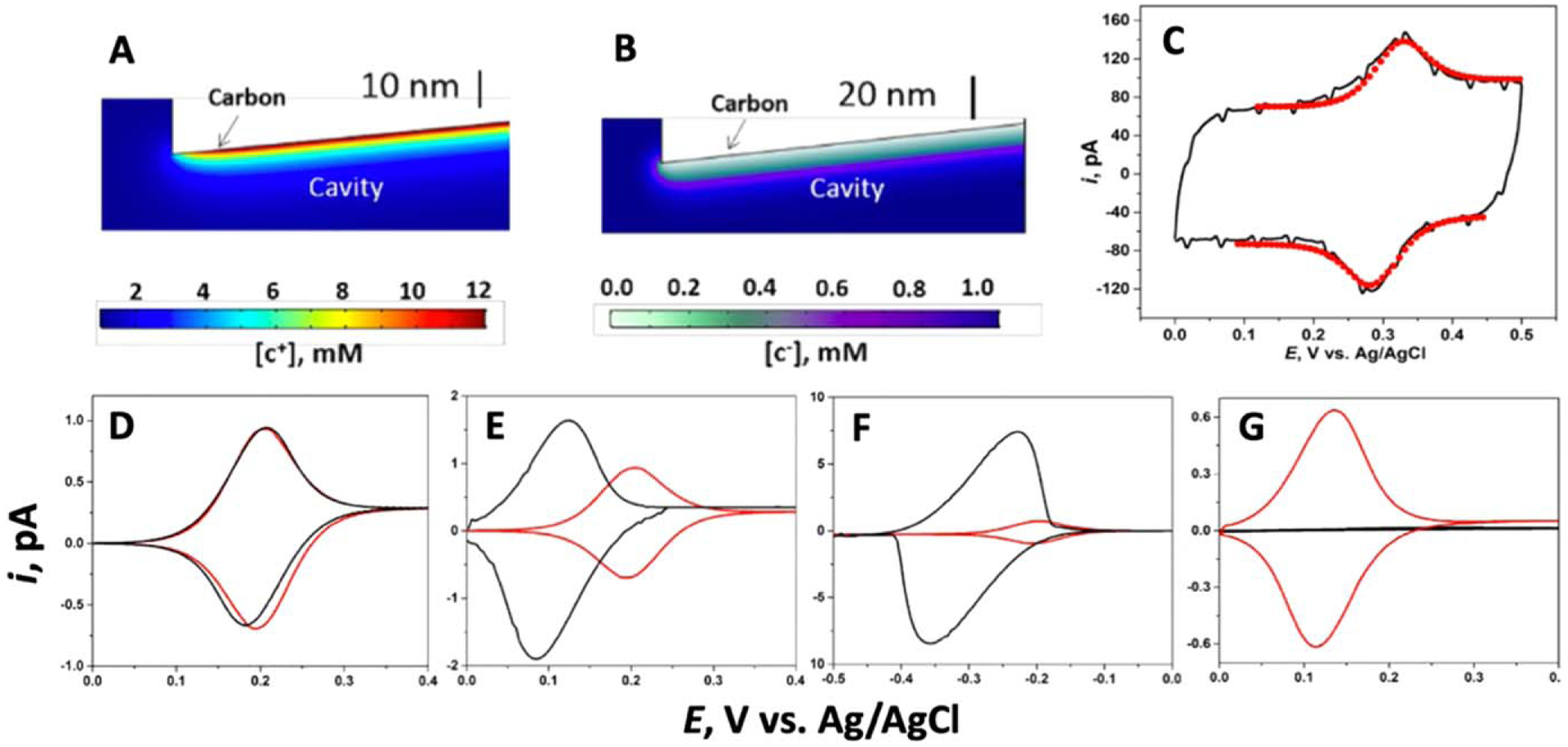
Simulated ion distribution near the charged carbon surface inside a 65 nm radius CNP: (a) cations, increased concentration ([c^+^] ≈ 12 mM); and (b) anions ([c^–^] ≈ 0 mM), almost completely depleted; the bulk value for the cations and anions in the outer solution is 1 mM. Excess electrolyte concentrations: (c) experimental (black solid line) and simulated (red dots) CVs at *v* = 20 V s^−1^ of 1 mM FcMeOH in 1 M KCl solution using a 65 nm radius CNP. Low electrolyte concentrations: CNP CVs of four different redox species with different charges and standard potentials (10 *μ*M) in 1 M KCl solution, (d) FcMeOH, (e) FcTMA^+^,(f) Ru(NH_3_)_6_^3+^, (g) Fe(CN)_6_^4–^. Adapted with permission from Ref. 76 Copyright 2019 American Chemical Society.

Bae et al. used CNPs to study permselective electrochemistry in a conductive nanopore.^76^ Due to the large conductive-surface-to-volume ratio as well as the nanometer-scale dimensions, nanopores and nanogaps have more significant electrical-double-layer (EDL) and surface-charge effects compared to macroscopic electrochemical systems. In a solution with low electrolyte concentration, cations were observed to accumulate, while anions depleted due to the negatively charged carbon surface and electric double-layer structure (Figs. 3a–3b). In high electrolyte concentrations, a quantitative fit was observed between simulated and experimental CNP CVs when there were weak double-layer effects, as shown in Fig. 3c. In low electrolyte concentrations, the simulations only provided semi-quantitative descriptions as more parameters were needed (Figs. 3d–3g). The CNPs can be more selective towards positively charged redox species at a low ionic concentration due to the negatively charged carbon surface, allowing the positively charged redox ions to accumulate. Alternatively, it is possible to reverse the permselectivity of the CNP based on the chemical modification of the carbon wall. Overall, the results would aid in the further development of CNP sensors for ionic analytes and sensitivity improvement.

pH sensing is an important application of ICP sensing. Liu et al. used thiol-maleimide Michael addition reactions to functionalize the surface of a nanoprobe with thiol groups. They immobilized cysteine on the surface of the nanopipette coated with maleimide to produce pH-sensitive ion current rectification.^83^ In another work with silane modification, the ratio of succinic anhydride and aminopropyl dimethyl ethyl ethoxy silane was varied during the process of silanization to record the pH response.^77^ Moreover, the optimization of concentration and silanization time was carried out to determine the rectification relationship of nanopipettes. The highest pH response was recorded with nanopipettes silanized with equal amounts of carboxylic group and amino group. The optimal concentration and silanization time came out to be 25 mM and 1 h respectively. The nanosensor showed good linearity in the pH range of 3–8, which is versatile enough to be used in biological and non-biological applications and development of nanosized pH detection devices.

Chen et al. demonstrated the detection of the activity of alkaline phosphatase with a nanopipette functionalized with *O*-phospho-Ltyrosine (p-Tyr).^58^ The modification of the nanopipette resulted in reduction of the negative surface area of the nanopipette followed by the alkaline phosphatase catalyzed dephosphorylation of the immobilized p-Tyr. A linear relation between alkaline phosphatase activity and ionic current was confirmed by an ANOVA test. Moreover, the excellent selectivity against interfering molecules was shown by a functionalized nanopipette with a detection limit of 0.1 mU ml^−1^.

## Application in Single Entity Analysis

Single entity study is important because a real system is usually heterogeneous, e.g., containing nanoparticles with different sizes and shapes.^84^ Nanoelectrodes have applications to the field of single entity analysis as their size allows them to reduce background noise that may overshadow the effect of a particle in larger electrodes. Previous reviews have looked at techniques underlying single particle analysis; however, this field continues to grow as its applications expand. We will be discussing studies from the past two years that introduce new information and rely on resistive pulse, bipolar electrochemical coupling, and surface enhanced Raman spectroscopy (SERS).

RP sensing is a popular way of characterizing size and surface charge of nanoparticles as well as sensing the geometry of the particles.^85^ Raveendran et al. demonstrated that glass nanopipettes can detect structural and geometrical variations of DNA origami structures via RP sensing.^78^ DNA origami are self-assembled arbitrary DNA structures. Four structures with the same charge and molecular weight but different geometry including two frames (F1 and F2) and two tile structures (T1 and T2) were investigated. Utilizing quartz nanopipettes (80–100 nm), they found that the tile and frame DNA origami structure peaks produced from translocation have “w” and “v” like peaks, respectively. The “w” shape originated from either the void near the center of the frame origami DNA that allowed current to flow freely for a short time during the translocation process, or from a tumbling effect.^85^ The amplitude of the ionic current allows for further distinction between the four structures. The ionic current amplitude for both T1(6511 pA) and T2 (7510 pA) is larger than F1 (3710 pA) and F2 (5915 pA), which is due to the differences in counterion distribution between both geometries. Differentiating between F1 and F2 can be achieved by looking at the lag time between the translocation peaks. F1’s RP peaks have short lag time due to their symmetrical void while F2’s asymmetrical void results in requiring more time to register its second peak. In another resistive pulse work, Wang et al. attached a DNA-tetrahedron to short-chain oligonucleotides for signal amplification, as they would otherwise be undetectable.^86^ When the target sequence is complementary to that of the DNA tetrahedron, a hairpin loop on the tetrahedron that was previously tense opens to increase surface area and amplify the resistive pulse signal. When it is not complementary, the DNA tetrahedron does not bind and the signal will stay relatively small.

**Figure 4.**
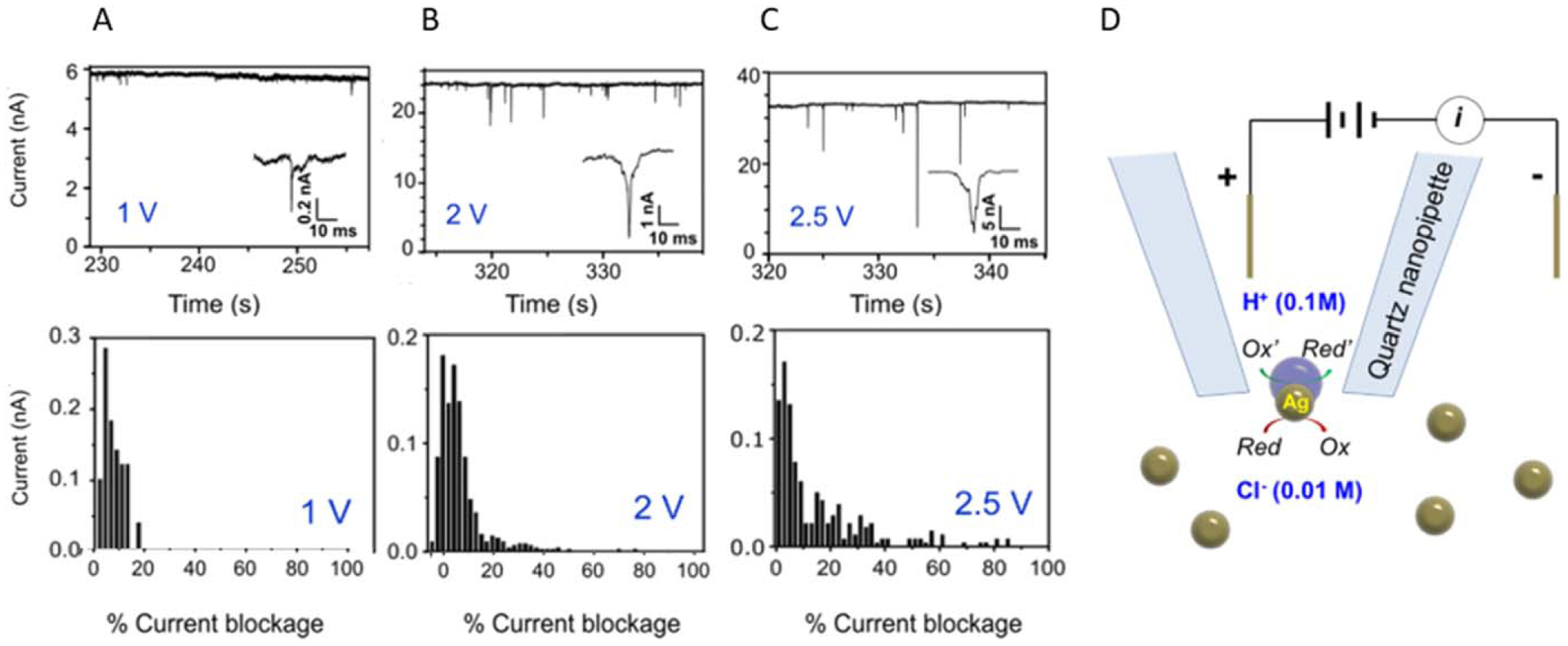
(a–c) From Han et al. depict Current-Time graphs and histograms of Current-%Current blockage at 1, 2, and 2.5 V respectively. (d) a schematic of their experimental setup and the reduction-oxidation reactions occurring on the silver nanoparticles being translocated. Adapted with permission from Ref. 79 Copyright 2019 American Chemical Society.

Han et al. worked to reduce noise caused by large applied voltages using quartz nanopipettes and resistive pulse sensing to observe the translocation of silver nanoparticles, as shown in Fig. 4.^79^ Previously, large voltages were required to initiate bipolar reactions over the particle length. However, nanopipettes can focus an applied voltage on the orifice of the pipette. By applying small voltages to conductive particles such as silver passing through the orifice, two redox reactions can be coupled on the two poles of the nanoparticle. These reactions are dependent on the voltage drop across the orifice being equal to or greater than the voltage requirement for the coupled reactions. They detected large current blockages that they attributed to the passing of single silver particles inducing coupled bipolar electrochemical reactions: the reduction of water inside the pipette and the oxidation of silver in the bulk solution. These large events were also due to the formation of a nanobubble with the release of H_2_ which increased the particle’s apparent size, blocking more of the pipette, and further demonstrating the coupling of the two reactions.

Lu et al. reported on monitoring hydrogen evolution reaction catalyzed by molybdenum sulfide (MoS_2_) Quantum dots using a glass nanopore confined probe.^80^ MoS_2_ Quantum dots were driven to the orifice of an 83 nm radius conical nanopore via electric field and electroosmotic flow to bind with silver nanoparticles to form a nanocomposite. The oxidation of silver nanoparticles forms a single nanoparticle electrode and induces the hydrogen evolution reaction catalyzed by the MoS_2_ Quantum dots leading to an increased ionic current measured via current-time traces. The effectiveness of the catalyst was monitored through the frequency of ionic blockages of the current via hydrogen bubbles formed by the reaction. In another coupling application of EC and RP, Pan et al used liposomes as a model system to demonstrate the capacity of CNPs in electrochemical RP sensing of redox species (e.g., ferrocyanide, dopamine and nitrite) contained in a single liposome.^53^ They loaded the liposomes with redox species K_4_[Fe(CN)_6_] and the RP recording showed both current blockages from the liposomes and upsurges from the oxidation of the redox species after the liposome’s collision with the carbon surface. The integrated current provides the information on the number of Fe(CN)_6_^4−^ ions within the vesicle. The same technique was successfully applied to dopamine and nitrite sensing, indicating its great potential in quantifying neurotransmitters and ROS/RNS stored in biological vesicles.

A novel application was introduced by combining a nanopipette sensor with surface enhanced Raman spectroscopy (SERS) for the detection of microplastic and nanoplastic particles.^81^ The nanosensor was fabricated by firstly depositing gold into a nanopipette through seed-mediated growth and then applying a strong voltage to form a nanopore with the gold electrode. The nanopore size (30–50 nm) was controlled by monitoring the resistance across the pore. The rectification phenomenon observed showed that the modified nanopipette is negatively charged. This fabrication is efficient and produces pipettes with a high SERS signal. Utilizing the model molecule R6G, translocation was proven to be electrochemically driven through the nanopore with the voltage of −1 V applied. In turn, the Raman microscope light records a Raman spectrum enhanced by the gold nanopore at the tip of the pipette. Nanoplastic spheres of 20 nm in size were characterized showing this method to be more sensitive than FTIR method which has a spatial resolution of 10–20 *μ*m.

## Conclusions and Future Prospects

In this review, we highlighted important progress in developing novel pre-pulled nanopipette-based-nanosensors for chemical and biological sensing. Four typical types of these nanosensors have been reviewed, which include open nanopipettes mainly for resistive pulse sensing, chemically modified nanopipettes that provides sensitive current rectification to selective analytes, nanoelectrodes that are either fully or partially filled with conductive materials for inducing electrochemical sensing of redox species, and wireless nanoelectrodes that couple with bipolar behavior. Built on pre-pulled nanopipettes that are needle-like and ultra-small in dimensions, these nanosensors benefit from the ease of fabrication, wide range of selections in modification for making multi-functional probes, and minimized overall dimension that enables detection of individual entities in low volume samples.

As technological advancements continue to be developed, the application of these nanosensors to the chemical and biological sensing will continue to expand. Finding easier and low-cost characterization methods, improving the fabrication reproducibility, and developing more rigid probes that are easy to handle would be necessary to expand the application. Another important direction would be further improving the sensing selectivity especially when it comes to intracellular studies, which could be achieved through specific chemical modification to the conductive surface and/or combining with novel functionalized nanomaterials.^87,88^ A better understanding of the relationship between the surface properties and transport processes in nanopipettes is also needed to enable the development of tuneable resistive-pulse or rectification sensors for specific analytes.^16^ In the field of resistive pulse sensing of single entities, it is necessary to establish a more detailed relationship between the current pulse and geometry of the entity to reveal the fine structures. Finally, utilizing the various geometries, such as nanochannels, nanopores, and nanocavities, allows one to develop new sensing mechanisms especially for the emerging interest in single molecule analysis,^23^ which is an exciting direction that requires further experimental and theoretical investigations.

## Figures and Tables

**Figure 1.**
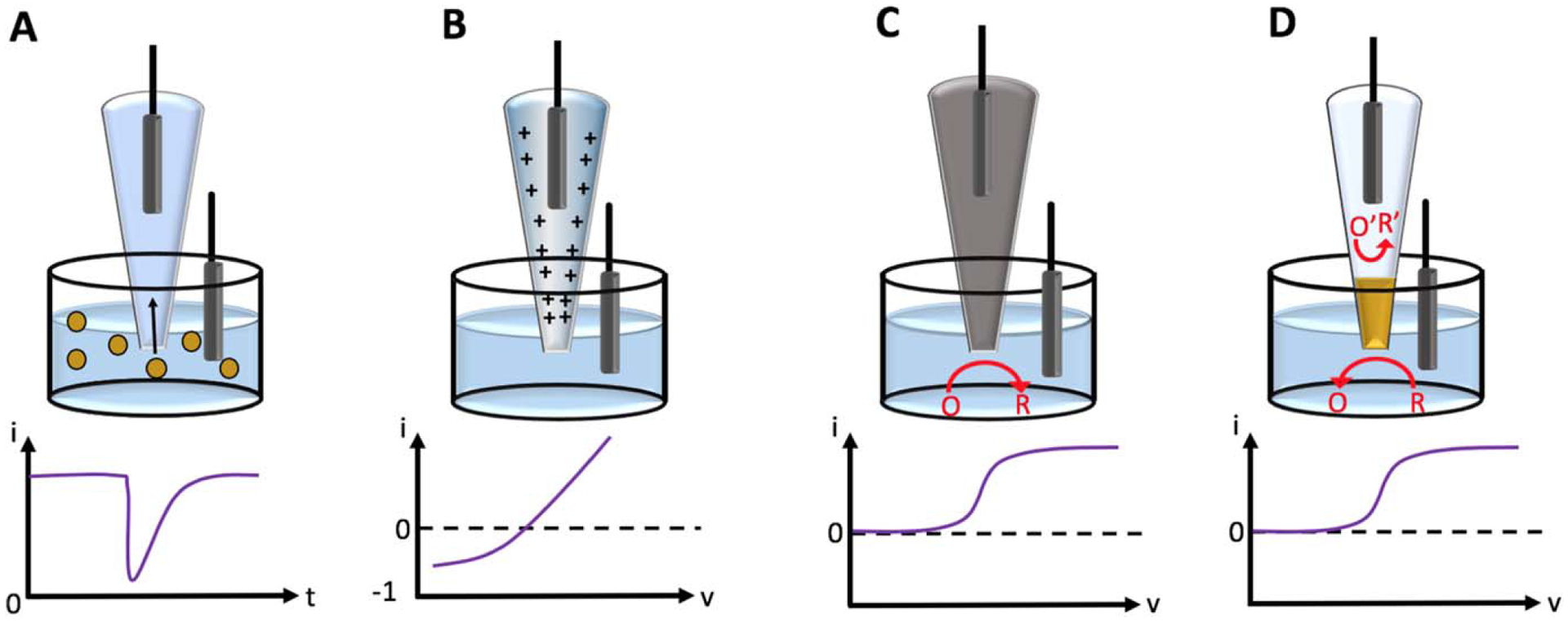
Schematic of different types of nanosensors built on pre-pulled nanopipettes. (a) an open bare nanopipette that senses nanoparticle through resistive pulse sensing; (b) a chemically modified nanopipette that establishes a strong current rectification behavior; (c) a fully-filled nanoelectrode that undergoes redox reaction at the orifice; and (d) a “wireless” nanoelectrode that establishes bipolar electrochemistry behavior.

**Table I.** Literature summary of applications of pre-pulled nanopipette based nanosensors in chemical and biological sensing within the last two years.

Sensor	Size (radius)	Technique	Medium/Sample	Analyte	Information	References
Platinized CNE	<100 nm	SECM Amperometry	Living macrophages in PBS	H_2_O_2_, ONOO^−^, NO^·^, NO_2_^−^	Production rates	47
Cytochrome c modified nanopipette	~20 nm	Linear sweep voltammetry	in vitro: PBS in vivo: breast epithelial cells	O_2_NO^·^	Linear range: 0.147 to 1.47 *μ*M; demonstration of in vivo sensing	62
Cavity CNP	100–200 nm	FSCV	Mouse brain slices	Dopamine	Demonstration of in vivo sensing	45
Cavity/openCNP	10–200 nm	CV	PBS	Dopamine	Detection limit: 100 pM	46
Platinized CNE	~66 nm	CV	in vitro: PBS in vivo: single lysosomes	Glucosidase activity (H_2_O_2_)	Demonstration of in vivo sensing	74
Cavity CNP	~95nm	CV	PB	Glucose	Detection limit: 0.016 mM; linear range: 0 to 15 mM; sensitivity: (16 ± 11) pA Mm^−1^	52
Nanopipette-ITIES	320–340 nm	CV	Water-HCl/DCE	GABA	Linear range: 0.25 to 1.0 mM; diffusion coefficient: 6.09 (±0.58) × 10^−10^m^2^s^−1^	75
CNP	20–200 nm	CV	KCl	FcMeOH, FcTMA^+^, Ru(NH_3_)_6_^3+^, Fe(CN)_6_^4−^	Detection limit: 10 pM for Ru(NH_3_)_6_^3+^	76
Silanized nanopipette	~45 nm	I-V	KCl	H^+^	Linear range: pH 3–8	77
APTES-modified nanopipette	~41 nm	I-V	PBS	Alkaline phosphatase activity	Detection limit: 0.1 mU ml^−1^	58
Nanopipette	40–50 nm	RP	KCl	DNA Origami	Differentiating geometry	78
Nanopipette	~30 nm	RP	Various ionic solutions	40 nm Ag nanoparticle	Demonstration of transient bipolar electrochemistry	79
MoS_2_ modified nanopipette	~41.5 nm	RP	Inside: H_2_SO_4_; Outside: KCl	H_2_	Demonstration of direct monitoring HER	80
CNP	125–250 nm	RP	Liposomes in PBS	K_4_[Fe(CN)_6_] dopamine nitrite	Demonstration of liposomes analysis	53
Gold nanopore pipette	15–25 nm	I-V, SERS	KCl	Nanoplastics	Detectable size range: a few to 30 nm	81
